# CT-guided transforaminal versus C-arm-guided intradiscal ozone injection for cervical disc herniation: a comparative cohort study

**DOI:** 10.3389/fmed.2026.1747595

**Published:** 2026-04-01

**Authors:** Mao Jiang Yang, Xian Qiong, Hongying Yang, Zhiqiang Qiu, Libing He, Hanfeng Yang, Xiaoxue Xu, Husni Ahmed Abdullah Al-Goshae

**Affiliations:** 1 Department of Pain, Affiliated Hospital of North Sichuan Medical College, Nanchong, Sichuan, China; 2 School of Graduate Studies, Post Graduate Centre, Management and Science University (MSU), Shah Alam, Selangor, Malaysia

**Keywords:** C-arm fluoroscopy, cervical disc herniation, computed tomography, intervertebral foramen, minimally invasive therapy, ozone

## Abstract

**Objective:**

This study compares a novel, disc-preserving minimally invasive technique targeting intraforaminal inflammation—CT-guided transforaminal ozone injection (Group A)—with the conventional intradiscal decompression approach using C-arm-guided injection (Group B) for the treatment of cervical disc herniation (CDH).

**Methods:**

A retrospective cohort study was conducted on 398 patients with CDH treated between January 2020 and June 2024. Patients were divided into two groups based on the treatment received: Group A (*n* = 200), who received CT-guided transforaminal injections targeting the inflamed nerve root, and Group B (*n* = 198), who received C-arm-guided intradiscal injections. Primary outcomes included the Visual Analogue Scale (VAS) for pain, the Neck Disability Index (NDI) for function, and overall efficacy assessed by the modified MacNab criteria. Secondary outcomes included procedural parameters and complication rates. Assessments were performed preoperatively and at 1 week, 1 month, 3 months, and 6 months post-procedure.

**Results:**

Both groups showed significant postoperative improvements in VAS and NDI scores (*p* < 0.05). However, from 1 month onward, Group A demonstrated significantly lower (better) VAS and NDI scores compared to Group B (*p* < 0.05). At the 6-month follow-up, the rate of “excellent” or “good” outcomes per MacNab criteria was significantly higher in Group A (93.0%) than in Group B (86.4%) (*p* = 0.026). Group A had a longer mean procedure time but significantly less radiation exposure (*p* < 0.001). Critically, no major complications, particularly discitis, occurred in Group A. In contrast, Group B had two cases (1.0%) of suspected discitis and a significantly higher rate of transient symptom exacerbation (*p* = 0.014).

**Conclusion:**

CT-guided transforaminal ozone injection, a technique predicated on targeting intraforaminal inflammation rather than conventional intradiscal decompression, is associated with better 6-month outcomes for CDH. It provides more effective pain relief and functional improvement while demonstrating an enhanced safety profile by preserving disc integrity and eliminating the risk of iatrogenic discitis. These findings establish this approach as a precise, safe, and highly promising minimally invasive treatment strategy for cervical radiculopathy.

## 1 Introduction

Cervical disc herniation (CDH) is a prevalent cause of neck-shoulder pain and upper limb radicular symptoms. Beyond typical radiculopathy, CDH is also a relevant cause of blurred vision, headache, tinnitus, nausea, gastrointestinal discomfort, hypomnesia, and palpitations, significantly impairing patients’ quality of life (1–4). Furthermore, CDH contributes to a substantial global economic burden, with spinal disorders estimated to cost tens of billions of dollars annually, underscoring the critical need for cost-effective and efficacious therapeutic strategies (5).

Clinical management strategies encompass a spectrum from conservative management to surgical interventions (6). However, a subset of patients responds inadequately to conservative therapy, while open surgical procedures are associated with significant trauma, prolonged recovery, and potential long-term complications. Consequently, the pursuit of effective, safe, and minimally invasive therapeutic approaches remains a central focus of clinical research.

Minimally invasive interventional techniques, particularly medical ozone (O_2_-O_3_) injection therapy, have emerged as important treatment modalities for disc herniation. This is attributed to ozone’s unique multi-faceted mechanisms of action, including potent anti-inflammatory and analgesic effects, as well as its ability to oxidize the nucleus pulposus, thereby reducing intradiscal pressure (7–9). Ozone effectively oxidizes and denatures proteoglycans within the nucleus pulposus, leading to dehydration and subsequent retraction of the herniated disc material, which alleviates mechanical compression on nerve roots. Simultaneously, it inhibits the release of inflammatory mediators such as phospholipase A2 (PLA2), resulting in powerful local anti-inflammatory effects (8).

In previous clinical practice, the image-guided anterior transdiscal approach has been a commonly employed technique. This method involves advancing the puncture needle into the intervertebral disc under imaging guidance. However, it presents significant limitations: the puncture inherently disrupts the integrity of the annulus fibrosus, potentially accelerating disc degeneration, and carries risks of inducing discogenic pain or infection (10–13). Building upon an evolving understanding of CDH pathophysiology—specifically, that symptoms arise not only from mechanical compression but also critically from sterile inflammation surrounding the affected nerve roots (14, 15)—we redirected the therapeutic focus from “intradiscal decompression” to “intraforaminal anti-inflammation.” We innovatively adopted a computed tomography (CT)-guided anterolateral approach, precisely targeting the intervertebral foramen region where nerve root compression and inflammatory reactions are most pronounced, rather than entering the disc space. The superior resolution of CT allows for clear visualization of anatomical structures, ensuring both the safety of the puncture trajectory and the accuracy of target localization. This enables the direct delivery of therapeutic agents to the inflammatory epicenter without causing damage to the intervertebral disc itself.

This retrospective study aimed to compare the clinical efficacy and safety of CT-guided anterolateral transforaminal ozone injection with the traditional C-arm fluoroscopy-guided intradiscal injection technique. The goal is to provide evidence for a novel approach to minimally invasive CDH treatment characterized by enhanced precision, safety, and therapeutic efficacy.

## 2 Methods

### 2.1 Study design and patients

This single-center, retrospective, non-randomized cohort study enrolled consecutive patients with CDH receiving ozone injection therapy at our institution between January 2020 and June 2024. Patients in Group B (C-arm-guided) were primarily treated during the earlier phase of our study period, predominantly from (January 2020) to (December 2022). With the subsequent introduction and standardization of the CT-guided transforaminal approach at our institution, patients in Group A (CT-guided) were treated predominantly from (January 2022) to (June 2024). Although there was an overlapping transition period, the two cohorts are largely sequential rather than strictly contemporaneous. To maximize statistical power, all eligible patients during this period were included without an *a priori* sample size calculation. Treatment allocation was based on the temporal evolution of clinical practice and patient preference rather than randomization. The study was approved by the Medical Ethics Committee of the Affiliated Hospital of North Sichuan Medical College (File: 2025ER355-1), and all patients provided written informed consent.

#### 2.1.1 Inclusion criteria

- Confirmed diagnosis of CDH based on clinical symptoms, physical signs, and magnetic resonance imaging (MRI).
- Primary clinical presentation of persistent neck-shoulder pain accompanied by radicular symptoms (pain, numbness) corresponding to the MRI-identified culprit level(s).
- Failure or insufficient response to at least 3 months of systematic conservative management (including pharmacotherapy, physical therapy, etc.).
- Age between 18 and 75 years.
- Agreement to undergo minimally invasive interventional therapy and ability to comply with postoperative follow-up.

#### 2.1.2 Exclusion criteria

- Concurrent cervical spinal stenosis, cervical instability, fracture, tumor, tuberculosis, or severe deformity.
- Presence of disc calcification or osteophyte formation at the herniated level causing bony compression/entrapment of the nerve root.
- Presence of myelopathic symptoms (cervical spondylotic myelopathy).
- Coagulation disorders, infection at the puncture site, or severe systemic diseases (e.g., cardiac, hepatic, pulmonary, renal).
- Hyperthyroidism or pregnancy.
- History of previous cervical spine surgery.
- Psychiatric disorders or inability to cooperate with the procedure or follow-up assessments.

**Figure 1:**
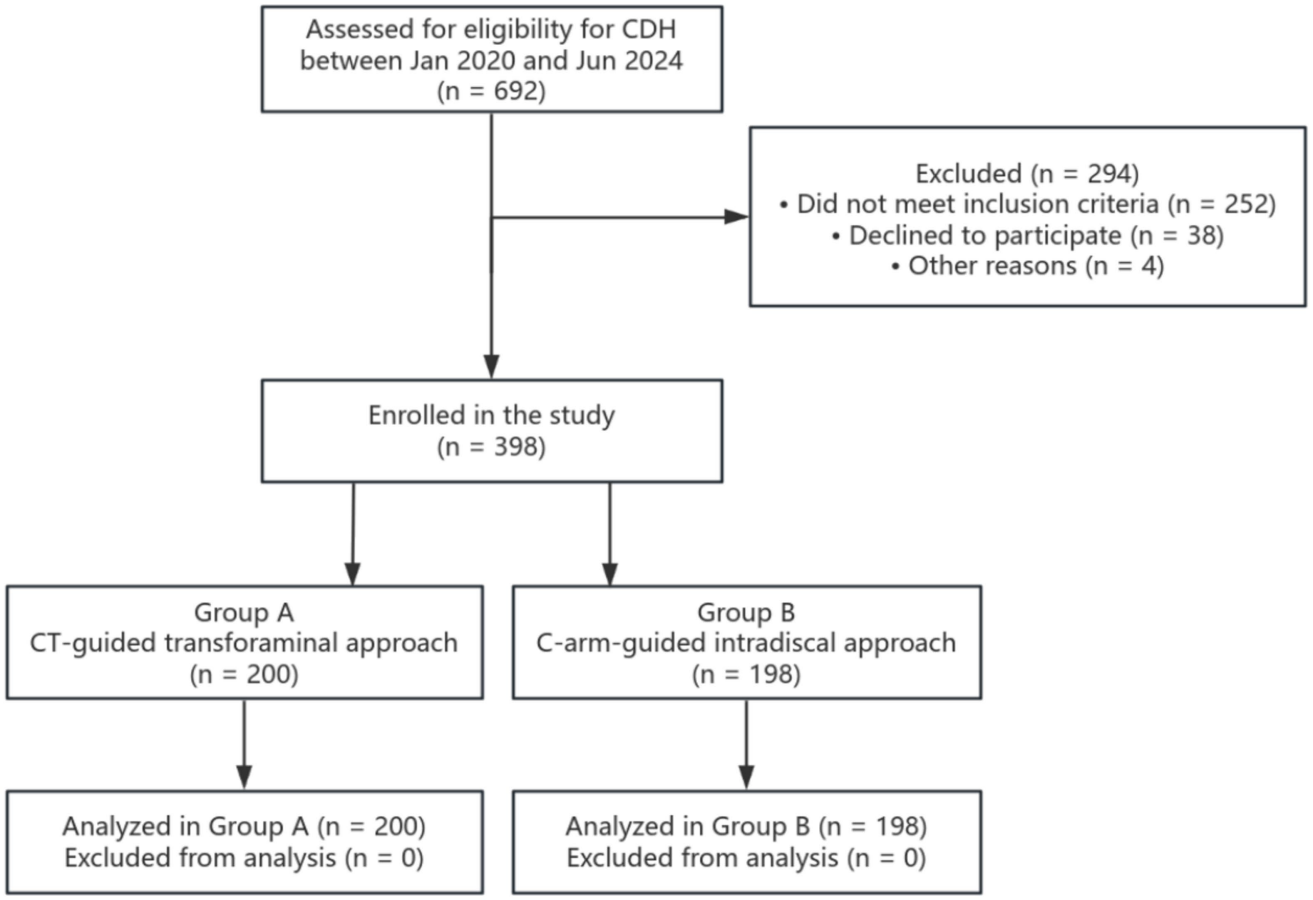
STROBE flow chart of patient enrollment and allocation. The diagram illustrates the study flow, detailing the initial eligibility assessment, exclusions, and final assignment to either the CT-guided transforaminal ozone injection cohort (Group A, *n* = 200) or the C-arm-guided intradiscal ozone injection cohort (Group B, *n* = 198). All enrolled patients were included in the final analysis.

Following the application of the inclusion and exclusion criteria, patient screening, enrollment, and treatment allocation were documented in accordance with the STROBE guidelines (Figure 1). Ultimately, a total of 398 eligible patients were enrolled and categorized into Group A (*n* = 200, receiving CT-guided transforaminal injection) and Group B (*n* = 198, receiving C-arm-guided intradiscal injection).

### 2.2 Surgical procedure

All procedures were performed by the same team of experienced physicians.

#### 2.2.1 C-arm guided group (anterior transdiscal approach)

**Figure 2:**
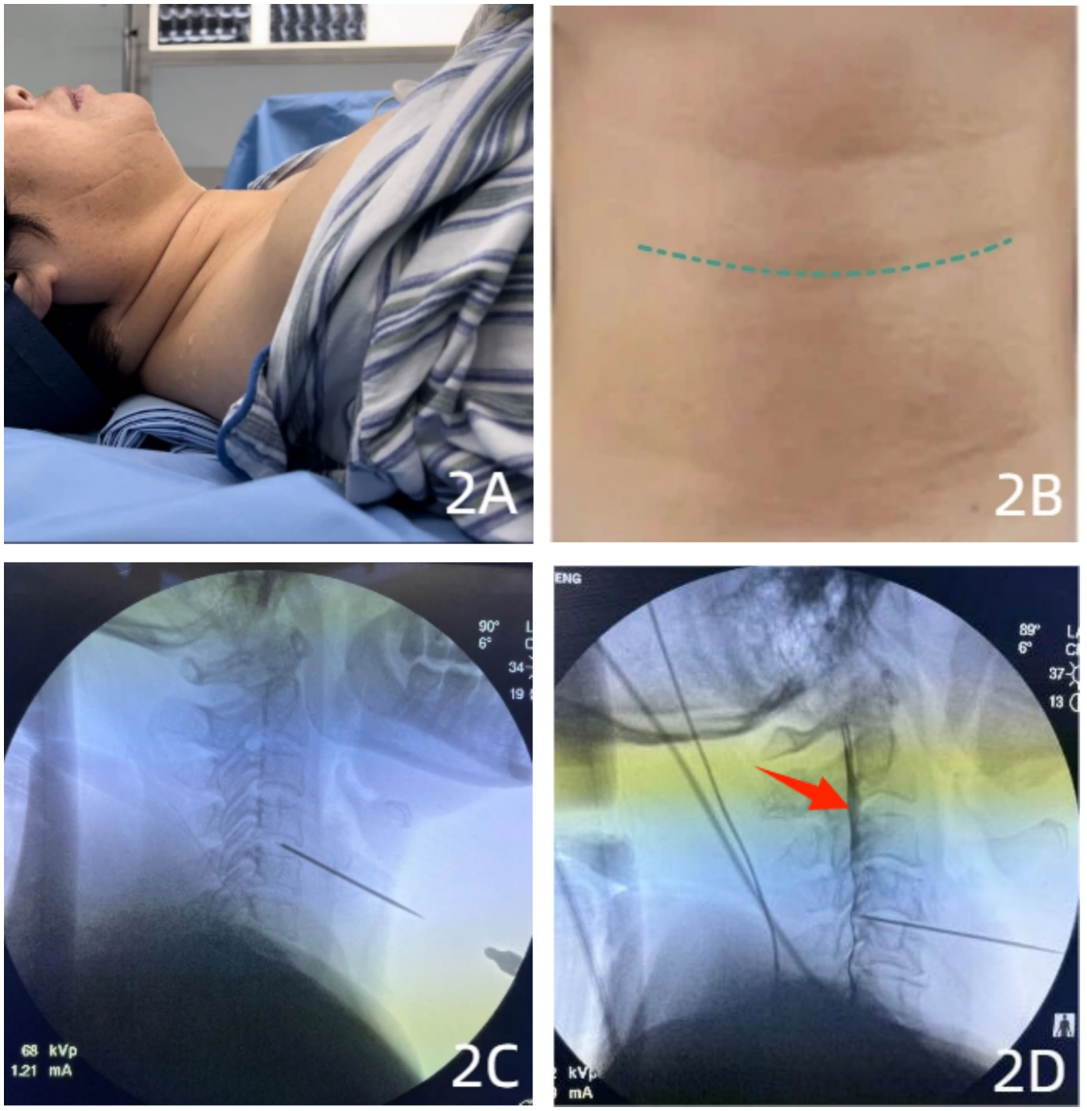
C-arm-guided intradiscal injection in a 68-year-old female patient. **(A)** Patient positioned supine with a thin pillow supporting the neck. **(B)** Surface landmark identification based on the second cervical skin crease. **(C)** Lateral fluoroscopic view confirming needle placement at the center of the intervertebral disc. **(D)** Injection of contrast medium, ozone, and anti-inflammatory solution, with contrast dispersion observed within the spinal canal (red arrow), indicating an annular tear.

All procedures were performed by the same team of experienced physicians. Patients were positioned supine with a thin pillow placed posteriorly beneath the neck to induce slight extension, using the second cervical skin crease (corresponding approximately to the C6 vertebral level) as the primary surface landmark. Following standard skin disinfection and draping, the target intervertebral disc space was localized and the skin entry point marked under C-arm fluoroscopy using both anteroposterior (AP) and lateral projections, typically medial to the sternocleidomastoid muscle border. The surgeon displaced the trachea, esophagus, and carotid sheath contralaterally using the index and middle fingers of the left hand, palpating the anterior border of the cervical vertebral body at the thyroid cartilage level. After local anesthesia infiltration (1% lidocaine), a 22-gauge coaxial puncture needle was advanced under intermittent AP and lateral fluoroscopic guidance, carefully avoiding vascular structures, until positioned within the nucleus pulposus of the target intervertebral disc. Correct intradiscal needle tip position was confirmed; aspiration ensured absence of blood or cerebrospinal fluid (CSF). Subsequently, 1 mL of contrast medium (iohexol) was slowly injected for discography, with real-time fluoroscopic observation of contrast morphology and any extravasation to confirm placement and assess annulus fibrosus integrity. Upon confirmation, therapeutic agents were injected sequentially: 5 mL of medical ozone (40 μg/mL), 5 mL of sterile normal saline, and 2 mL of an anti-inflammatory analgesic solution (containing 0.5% lidocaine 1 mL, compound betamethasone 1 mL, and sterile water for injection 1 mL). The needle was withdrawn, manual compression applied to the puncture site, and an adhesive bandage applied (Figure 2).

#### 2.2.2 CT-guided group (anterolateral transforaminal approach)

**Figure 3:**
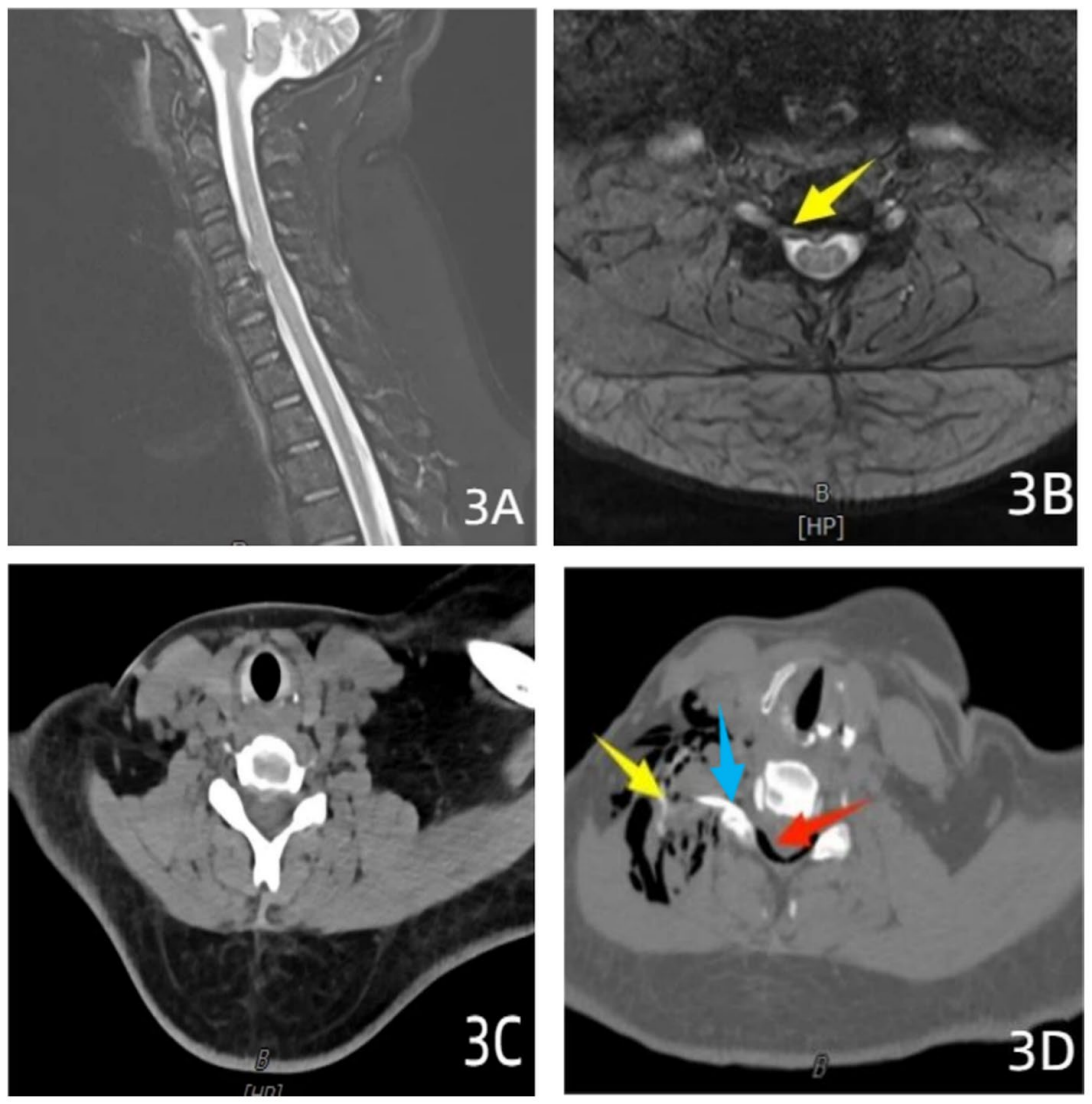
CT-guided transforaminal injection in a 53-year-old male patient. **(A)** Sagittal T2-weighted fat-suppressed MR image demonstrating C5/6 disc herniation compressing the anterior margin of the dural sac. **(B)** Axial T2-weighted fat-suppressed MR image revealing right-sided foraminal-type disc herniation at C5/6 (yellow arrow). **(C)** Axial CT scan confirming disc herniation without calcification. **(D)** CT-guided right transforaminal injection of ozone combined with anti-inflammatory solution, with red arrows indicating ozone distribution posterior to the dural sac and within soft tissue spaces, and yellow arrow indicating contrast medium extravasation. The blue arrow indicates that the tip of the needle is located at the anterior border of the facet joint.

All procedures were performed by the same team of experienced physicians. Patients were positioned supine with the head rotated approximately 15–30° towards the contralateral side to optimize exposure of the affected intervertebral foramen. Thin-slice (1.0 mm) scanning using a 128-slice spiral CT scanner localized the target intervertebral foramen, with the anterior margin of the superior articular process designated as the target. The optimal puncture trajectory was meticulously planned on CT images to avoid the carotid sheath, thyroid gland, trachea, esophagus, and vertebral artery; the skin entry point was typically anterior to the sternocleidomastoid muscle border, with a puncture angle of approximately 45–60° relative to the sagittal plane. Following marking of the entry point, standard skin disinfection, draping, and local anesthesia (1% lidocaine) were administered. Using intermittent CT guidance, a 22-gauge coaxial puncture needle was advanced along the pre-planned trajectory and angle, adhering strictly to a “puncture-scan-adjust” protocol to progressively guide the needle tip precisely into the target intervertebral foramen region. The ideal final needle position was confirmed at the lateral aperture of the intervertebral foramen, ventral to the exiting nerve root and anterior to the facet joint, ensuring optimal therapeutic agent diffusion around the nerve root while maintaining safe distance from the vertebral artery and dural sac. After confirming satisfactory needle position and aspirating to exclude blood or cerebrospinal fluid (CSF), 1 mL of contrast medium was slowly injected under real-time CT monitoring to observe its dispersion within the spinal canal and definitively exclude intravascular or intrathecal placement. Subsequently, an identical volume and concentration of ozone, sterile normal saline, and anti-inflammatory analgesic solution as used in Group B was injected. The needle was withdrawn, manual compression applied for hemostasis, and the site covered with a sterile dressing (Figure 3).

### 2.3 Postoperative management

All patients in both groups were required to maintain bed rest for 6 h postoperatively for observation of potential adverse reactions. A cervical collar was worn for 2 weeks postoperatively. Oral neurotrophic medication (e.g., mecobalamin 0.5 mg three times daily) and non-steroidal anti-inflammatory drugs (NSAIDs, e.g., celecoxib 0.1 g twice daily) were prescribed for 3 days. Patients were instructed to avoid strenuous neck movements and heavy lifting for the first postoperative month.

### 2.4 Outcome measures and efficacy assessment

#### 2.4.1 Baseline characteristics

Data on patient gender, age, disease duration, body mass index (BMI), smoking history, and distribution of culprit levels were recorded and compared between the two groups.

#### 2.4.2 Procedure-related parameters

Puncture success rate, single-session procedure time (defined as the duration from local anesthesia infiltration to needle withdrawal), and radiation exposure parameters were recorded. For Group A (CT-guided), the effective radiation dose was calculated based on the total mAs from all scans performed during the procedure and converted to millisieverts (mSv). For Group B (C-arm-guided), the total fluoroscopy time (seconds) was recorded.

#### 2.4.3 Clinical outcome assessment

a) Pain intensity: Assessed using the Visual Analogue Scale (VAS), where 0 represents no pain and 10 indicates excruciating pain (16).
b) Cervical function: Evaluated using the Neck Disability Index (NDI) questionnaire. The total NDI score is 50 points, with higher scores indicating greater functional impairment. Scores were converted to a percentage (Score/50 × 100%) for analysis (17).
c) Overall clinical efficacy: Assessed using the modified MacNab criteria.

- Excellent: Complete resolution of symptoms, return to normal work and activities.
- Good: Minimal residual symptoms, occasional mild discomfort not interfering with work or activities.
- Fair: Partial symptom relief, activity limitations, continued need for medication.
- Poor: No improvement or worsening of symptoms.

The excellent/good rate was calculated as (Number of Excellent + Number of Good)/Total Number × 100%.

d) Complications: All intraoperative and postoperative complications were recorded and compared between groups. These included transient exacerbation of radicular symptoms, hematoma, infection, hoarseness, dysphagia, discitis, and others.

All assessments (VAS, NDI, MacNab) were performed preoperatively and at 1 week, 1 months, 3 months, and 6 months postoperatively by a third-party researcher blinded to the surgical group assignment.

### 2.5 Statistical analysis

Statistical analyses were performed using SPSS software (version 27.0). Continuous variables conforming to a normal distribution are presented as mean ± standard deviation (SD) and were compared between groups using independent samples *t*-tests. Non-normally distributed continuous data were analyzed using the Mann–Whitney *U* test. Categorical variables are presented as number (percentage) and were compared using the chi-square test or Fisher’s exact test, as appropriate. Repeated-measures analysis of variance (RM-ANOVA) was used to analyze the longitudinal changes in VAS and NDI scores across follow-up time points. All tests were two-tailed, and a *p*-value <0.05 was considered statistically significant. Data visualization and graphical representations were generated using R software (version 4.5.1, R Foundation for Statistical Computing, Vienna, Austria) with the ggplot2 package.

## 3 Results

### 3.1 Comparison of baseline characteristics

A total of three hundred and ninety-eight (398) patients were enrolled in this study, with 200 patients assigned to Group A and 198 to Group B. No statistically significant differences were observed between the two groups regarding gender distribution, age, disease duration, body mass index (BMI), smoking history, history of hypertension (HTN) or diabetes mellitus (DM), or distribution of the affected disc levels (all *p* > 0.05). Furthermore, preoperative Visual Analogue Scale (VAS) and Neck Disability Index (NDI) scores showed no significant intergroup differences (*p* > 0.05). Detailed baseline characteristics are presented in Table 1.

**Table 1:** Comparison of baseline characteristics between groups [mean ± SD; *n* (%)].

Characteristic	Group A (*n* = 200)	Group B (*n* = 198)	*t*/*χ^2^*	*p*
Gender (male/female)	108/92	102/96	0.284	0.594
Age (year)	48.5 ± 10.2	49.1 ± 9.8	−0.621	0.535
Duration (year)	15.2 ± 8.5	14.8 ± 9.1	0.467	0.641
BMI (kg/m^2^)	24.1 ± 3.3	24.5 ± 3.6	−1.168	0.244
Smoking (%)	62 (31.0)	55 (27.8)	0.697	0.404
Hypertension (%)	45 (22.5)	40 (20.2)	0.384	0.535
Diabetes (%)	22 (11.0)	18 (9.1)	0.473	0.492
Herniated level(s)			1.872	0.392
C3/4	14 (7.0)	12 (6.1)		
C4/5	42 (21.0)	38 (19.2)		
C5/6	110 (55.0)	112 (56.6)		
C6/7	48 (24.0)	48 (24.2)		
VAS	7.6 ± 0.9	7.5 ± 1.0	1.055	0.292
NDI	45.8 ± 8.2	46.3 ± 7.9	−0.634	0.526

BMI, body mass index; VAS, Visual Analogue Scale; NDI, Neck Disability Index. The cohorts demonstrated comparable baseline profiles across all measured parameters (*p* > 0.05), ensuring valid intergroup comparisons.

### 3.2 Comparison of procedure-related parameters and clinical efficacy

All punctures were successfully completed on the first attempt in both groups. The mean procedure time was significantly longer in Group A (19.5 ± 4.2 min) compared to Group B (14.8 ± 3.5 min) (*p* < 0.001). In contrast, Group A demonstrated a significantly lower mean effective radiation dose than Group B, indicating a substantial reduction in radiation exposure (*p* < 0.001) (see Table 2).

**Table 2:** Comparison of procedure-related parameters between groups (mean ± SD).

Parameter	Group A (*n* = 200)	Group B (*n* = 198)	*t*	*p*
Surgical success rate (%)	100 (200/200)	100 (198/198)	N/A^b^	N/A^b^
Puncture time (min)	19.5 ± 4.2	14.8 ± 3.5	12.145	<0.001
Radiation dose (mSv)^a^	0.8 ± 0.3	1.6 ± 0.5	−18.966	<0.001

N/A, not applicable (puncture success rate was 100% in both groups).

a Effective radiation dose is expressed in millisieverts (mSv).

b Procedure time was significantly longer in Group A compared to Group B (*p* < 0.001), while radiation exposure dose was significantly lower in Group A compared to Group B (*p* < 0.001).

### 3.3 Postoperative pain and functional improvement

Postoperatively, both groups demonstrated significant reductions in Visual Analogue Scale (VAS) pain scores and Neck Disability Index (NDI) scores at all follow-up time points compared to preoperative values (*p* < 0.001). Detailed data are presented in Table 3.

**Figure 4:**
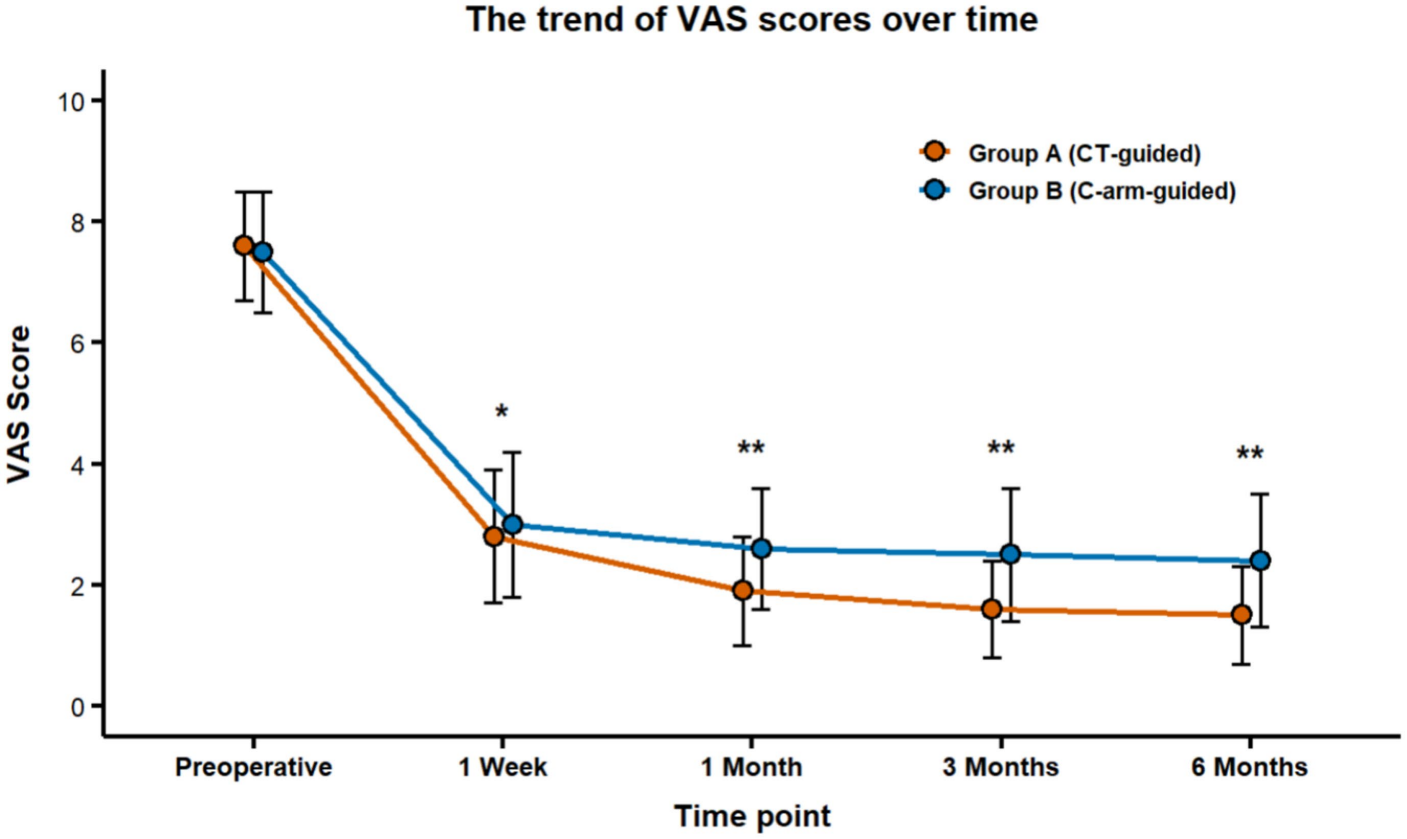
Temporal changes in neck/arm pain VAS scores following operation. Statistical significance was defined as *p* < 0.05. ^*^p < 0.05 vs. preoperative baseline (indicated by single asterisk). ^**^p < 0.05 vs. Group B at the same time point (indicated by double asterisks). VAS, Visual Analogue Scale.

a) VAS score changes.

- Short-term effect: At 1 week postoperatively, VAS scores decreased significantly in both groups (Group A: 2.8 ± 1.1 vs. Group B: 3.0 ± 1.2), with no statistically significant difference observed between groups (*p* > 0.05).
- Mid-to-long term effects: Beginning at the 1-month follow-up, Group A consistently exhibited significantly lower VAS scores than Group B (*p* < 0.05). By 12 months postoperatively, the mean VAS score in Group A (1.5 ± 0.8) was significantly better than that in Group B (2.4 ± 1.1) (*p* < 0.001) (Figure 4).

**Table 3:** Comparison of VAS and NDI scores between groups at preoperative and postoperative time points (mean ± SD).

Time point	VAS		NDI	
	Group A (*n* = 200)	Group B (*n* = 198)	Group A (*n* = 200)	Group B (*n* = 198)
Preoperative	7.6 ± 0.9	7.5 ± 1.0	45.8 ± 8.2	46.3 ± 7.9
Post-1 week	2.8 ± 1.1^a^	3.0 ± 1.2^a^	22.5 ± 6.1^a^	23.8 ± 6.5^a^
Post-1 month	1.9 ± 0.9^b^	2.6 ± 1.0^a^	15.1 ± 5.8^b^	20.5 ± 7.0^a^
Post-3 months	1.6 ± 0.8^b^	2.5 ± 1.1^a^	13.2 ± 5.5^b^	19.5 ± 7.3^a^
Post-6 months	1.5 ± 0.8^b^	2.4 ± 1.1^a^	12.4 ± 5.6^b^	18.9 ± 7.2^a^

a Significant improvement compared to preoperative value within the same group (*p* < 0.05).

b Significantly better than Group B at the same time point (*p* < 0.05); VAS and NDI scores showed significant reductions at all postoperative time points compared to preoperative values (*p* < 0.001). Beginning at the 1-month follow-up, both VAS and NDI scores were significantly lower in Group A compared to Group B (*p* < 0.05).

**Figure 5:**
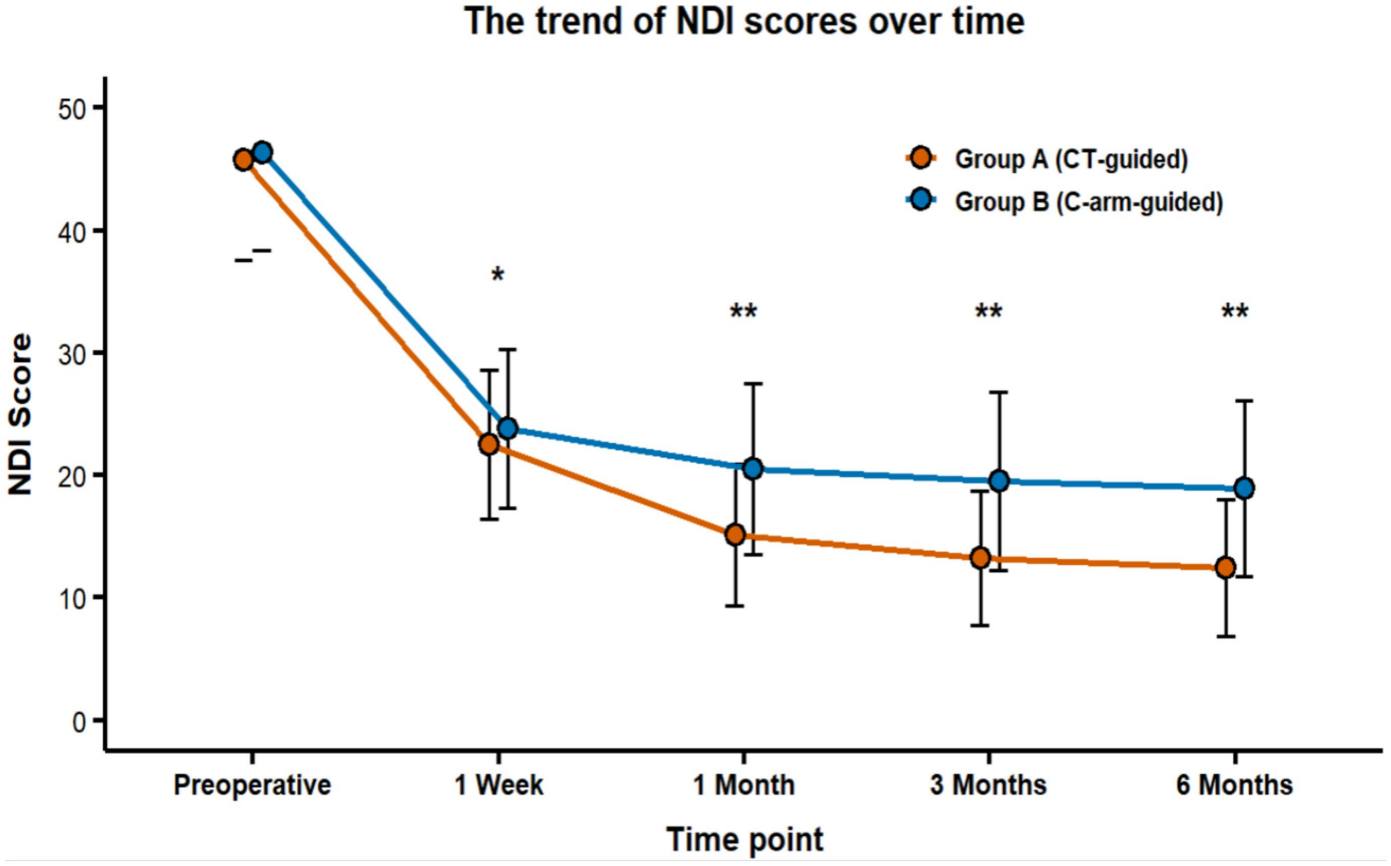
Temporal changes in NDI scores following operation. Statistical significance was defined as *p* < 0.05. ^*^p < 0.05 vs. preoperative baseline (indicated by single asterisk). ^**^p < 0.05 vs. Group B at the same time point (indicated by double asterisks). NDI, Neck Disability Index.

b) NDI score changes.

c Functional recovery trend: The trend in NDI score improvement paralleled that of the VAS scores. Group A demonstrated significantly lower NDI scores than Group B at the 1-month, 3-month, and 6-month follow-ups (all *p* < 0.05).
d Final assessment: At the 6-month assessment, the mean NDI score was 12.4 ± 5.6 in Group A compared to 18.9 ± 7.2 in Group B, representing a significant difference (Figure 5).

### 3.4 Comparison of overall clinical efficacy

Overall clinical efficacy was assessed at the final follow-up (6 months postoperatively) using the modified MacNab criteria. The excellent/good rate was 93.0% (186/200) in Group A (Excellent: 152 cases; Good: 34 cases) and 86.4% (171/198) in Group B (Excellent: 128 cases; Good: 43 cases). The difference in the excellent/good rate between the two groups was statistically significant (*χ*^2^ = 4.956, *p* = 0.026) (Table 4).

**Table 4:** Comparison of modified MacNab outcomes at 12 months postoperatively between groups [*n* (%)].

Outcome	Group A (*n* = 200)	Group B (*n* = 198)	*χ* ^2^	*p*
Excellent	152 (76.0)	128 (64.6)		
Good	34 (17.0)	43 (21.7)		
Fair	11 (5.5)	20 (10.1)		
Poor	3 (1.5)	7 (3.5)		
Excellent/Good Rate	186 (93.0)	171 (86.4)	4.956	0.026

Efficacy assessment based on modified MacNab criteria. Excellent/Good Rate = (Number of Excellent + Number of Good)/Total Number × 100%. The excellent/good rate was significantly higher in Group A (93.0%) compared to Group B (86.4%) (*p* = 0.026).

### 3.5 Comparison of postoperative complications

The overall complication rate was lower in Group A (1.5%) compared to Group B (4.0%), although this difference did not reach statistical significance (*p* = 0.175). In Group B, two cases (1.0%) of suspected discitis were identified based on recurrent severe neck pain 1 week postoperatively, accompanied by elevated CRP and ESR levels and MRI findings consistent with endplate edema. Both cases were successfully managed conservatively with intravenous antibiotics and immobilization, resolving without surgical intervention. Additionally, six patients (3.0%) in Group B developed transient exacerbation of neck stiffness and pain, likely attributable to increased intradiscal pressure during injection, which resolved spontaneously within 1 week. Group A had no severe complications such as infection or nerve injury. Only three patients (1.5%) exhibited minor subcutaneous ecchymosis at the puncture site, requiring no specific intervention. Neither group experienced hoarseness, dysphagia, or permanent neurological deterioration. Detailed complication data are presented in Table 5.

**Table 5:** Comparison of postoperative complications between groups [*n* (%)].

Complication type	Group A (*n* = 200)	Group B (*n* = 198)	*p*
Major complications	0 (0.0)	2 (1.0)	0.248
Discitis	0 (0.0)	2 (1.0)	
Permanent nerve root injury	0 (0.0)	0 (0.0)	
Vertebral artery injury/Hematoma	0 (0.0)	0 (0.0)	
Minor complications	3 (1.5)	6 (3.0)	0.342
Exacerbation of radicular symptoms	0 (0.0)	6 (3.0)	0.014
Local puncture site hematoma	3 (1.5)	0 (0.0)	0.124
Hoarseness/Dysphagia	0 (0.0)	0 (0.0)	
Total complication rate	3 (1.5)	8 (4.0)	0.175

Major complications were defined as those requiring additional medical intervention or hospitalization. *p*-values were calculated using the chi-square test or Fisher’s exact test, as appropriate. The difference in the total complication rate was not statistically significant (*p* = 0.175). Differences in major complication rate (*p* = 0.248) and minor complication rate (*p* = 0.342) were also not statistically significant. The incidence of transient exacerbation of radicular symptoms was significantly higher in Group B compared to Group A (*p* = 0.014).

## 4 Discussion

This study provides the first direct comparison of clinical outcomes between CT-guided anterolateral transforaminal ozone injection and conventional C-arm-guided anterior transdiscal ozone injection for treating cervical disc herniation (CDH). Our findings demonstrate that both techniques effectively alleviate pain and improve cervical function. However, the CT-guided transforaminal approach demonstrated significant advantages in sustained efficacy at the 6-month follow-up, functional recovery, and procedural safety.

### 4.1 Mechanisms underlying superior efficacy

The core innovation of this study lies in shifting the therapeutic target: from intradiscal decompression to intraforaminal anti-inflammation. The theoretical basis of traditional intradiscal injection is nucleus pulposus dehydration and shrinkage via ozone oxidation, thereby relieving mechanical nerve root compression (9, 18). This mechanism is undoubtedly effective, as evidenced by the >85% excellent/good rate in Group B. However, growing evidence indicates that radicular symptoms in CDH result from a combination of mechanical compression and chemical irritation (sterile inflammation) (14, 15, 18). Herniated nucleus pulposus releases pro-inflammatory mediators, such as tumor necrosis factor-alpha (TNF-α) and interleukin-1 beta (IL-1β), which directly irritate nerve roots, leading to edema, demyelination, and pain (19, 20).

Our modified CT-guided transforaminal technique directly addresses this inflammatory pathology. Via the anterolateral approach, ozone and anti-inflammatory solution are precisely delivered to the culprit zone surrounding the nerve root within the intervertebral foramen—the site of maximal compression and inflammatory concentration. Here, ozone exerts potent local anti-inflammatory effects, neutralizing inflammatory mediators, while also improving microcirculation, enhancing tissue oxygenation, and promoting resolution of neural edema (21–23). The adjunctive low-dose corticosteroid and local anesthetic further enhance anti-inflammatory and analgesic effects, blocking pain signal transmission (24, 25).

Although both groups showed similar short-term (1-week) improvement, likely attributable to the immediate effects of local anesthetic and steroid, Group A demonstrated significantly superior VAS and NDI scores from 3 months onwards. We attribute this sustained advantage to several key factors:

a) Enhanced target specificity: Group A’s direct delivery to the inflammatory epicenter provides potent, localized action, disrupting the pain vicious cycle more effectively and durably. In contrast, Group B’s intradiscal injection relies on passive drug diffusion through annular tears to reach the nerve root, resulting in unpredictable drug concentration and distribution at the target site.
b) Preservation of disc integrity: The transforaminal technique completely avoids disc puncture. This minimizes iatrogenic trauma and crucially preserves annular integrity and disc biomechanics. Evidence suggests any invasive disruption of the annulus fibrosus may accelerate disc degeneration (11–13). The annular violation inherent in Group B’s technique may underlie its slightly inferior long-term functional recovery (NDI scores).
c) Comprehensive therapeutic action: The transforaminal approach holistically addresses both key pathophysiological components: chemical irritation (via potent local anti-inflammation) and mechanical compression (by reducing neural edema). The intradiscal approach primarily targets mechanical decompression, with a weaker direct effect on the inflammatory cascade.

### 4.2 Safety advantages and technical considerations

Safety is paramount in minimally invasive therapies. In this study, Group A demonstrated superior safety, with no major complications such as discitis. This advantage stems directly from its “non-intradiscal” nature, fundamentally eliminating the risk of introducing bacteria into the poorly vascularized and infection-prone disc space (26, 27). The two discitis cases in Group B, while successfully treated, underscore the inherent risk of intradiscal procedures.

The primary strength of CT guidance lies in its unparalleled visualization of complex anatomy. The anterior cervical region contains densely packed critical structures – carotid sheath, vertebral artery, nerve roots, esophagus, and trachea. C-arm fluoroscopy, limited by its 2D imaging, cannot reliably distinguish these soft tissues, introducing uncertainty and reliance on operator anatomical knowledge and tactile feedback. Conversely, CT guidance enables: pre-procedural planning of an optimal, safe trajectory; real-time monitoring of needle tip position relative to vital structures during advancement. This facilitates true visualization-guided precision targeting, substantially mitigating the risk of inadvertent injury (28).

Technical considerations for CT guidance warrant discussion. Group A exhibited longer procedure times, primarily attributable to the requirements for CT scanning, image reconstruction, trajectory planning, and the inherent stepwise ‘scan-adjust-advance’ protocol, which is less fluid than real-time C-arm fluoroscopy; however, this time difference is anticipated to diminish with accumulated operator experience and optimized workflows. Regarding radiation exposure, while a single CT scan delivers a higher dose than a single C-arm fluoroscopic image, our strict adherence to the ALARA (as low as reasonably achievable) principle—utilizing low-dose protocols and minimizing scan frequency—resulted in a significantly lower total effective radiation dose for Group A compared to Group B (*p* < 0.001). This suggests that well-optimized CT guidance can achieve lower overall exposure than complex C-arm procedures necessitating extensive, multi-angle fluoroscopy, though future studies should conduct precise dosimetric comparisons.

### 4.3 Limitations and future perspectives

Several limitations must be acknowledged. First, the retrospective, single-center design carries inherent selection bias and provides a lower level of evidence. Crucially, without multivariable adjustment for potential confounders (e.g., disease duration, smoking history, comorbidities), our findings demonstrate an association rather than a definitive causal effect. Second, the sequential transition to CT guidance at our institution created non-contemporaneous cohorts, introducing potential chronological bias and learning curve effects that may favor the later CT group. Third, the 6-month follow-up precludes conclusions regarding long-term (>1 year) efficacy.

Despite these limitations, our findings highlight the clinical potential of the CT-guided transforaminal approach for CDH. Future prospective, randomized controlled trials (RCTs) are imperative to confirm these results. Such studies should ideally incorporate objective neural assessments (e.g., electromyography, high-resolution MRI) and seek to optimize specific therapeutic parameters.

## 5 Conclusion

In conclusion, CT-guided transforaminal ozone injection is associated with superior pain relief and functional improvement at 6 months compared to the C-arm-guided intradiscal approach. While demonstrating an enhanced safety profile, these findings require confirmation through prospective randomized trials.

## Data Availability

The original contributions presented in the study are included in the article/supplementary material, further inquiries can be directed to the corresponding authors.

## References

[1] Editorial Board of Chinese Journal of Surgery. The experts consensus on the classification, diagnosis and non-surgical treatment of cervical spondylisis(2018). Zhonghua Wai Ke Za Zhi. (2018) 56:401–2. doi: 10.3760/cma.j.issn.0529-5815.2018.06.001, 29886658

[2] Wong JJ Côté P Quesnele JJ Stern PJ Mior SA. The course and prognostic factors of symptomatic cervical disc herniation with radiculopathy: a systematic review of the literature. Spine J. (2014) 14:1781–9. doi: 10.1016/j.spinee.2014.02.032, 24614255

[3] Uysal A Güntel M. Evaluation of depression, anxiety and sleep quality scores in patients with cervical disc herniation: a study conducted in Turkey. Batı Karadeniz Tıp Dergisi. (2023) 7:31–7. doi: 10.29058/mjwbs.1220876

[4] Leung KKY Chu EC Chin WL Mok STK Chin EWS. Cervicogenic visual dysfunction: an understanding of its pathomechanism. Med Pharm Rep. (2023) 96:16–9. doi: 10.15386/mpr-2507, 36818319 PMC9924804

[5] Waheed MA Hasan S Tan LA Waheed MA-A Bosco A Reinas R . Cervical spine pathology and treatment: a global overview. J Spine Surg. (2020) 6:340–50. doi: 10.21037/jss.2020.01.12, 32309671 PMC7154356

[6] Wu PH Kim HS Jang IT. Intervertebral disc diseases PART 2: a review of the current diagnostic and treatment strategies for intervertebral disc disease. Int J Mol Sci. (2020) 21:2135. doi: 10.3390/ijms21062135, 32244936 PMC7139690

[7] Rybaczek M Mariak Z Grabala P Łysoń T. Minimally invasive percutaneous techniques for the treatment of cervical disc herniation: a systematic review and meta-analysis. J Clin Med. (2025) 14:3280. doi: 10.3390/jcm14103280, 40429275 PMC12112353

[8] Ghatge SB Asarkar A Warghade SS Shirsat S Deb A. Ozone disc nucleolysis for cervical intervertebral disc herniation: a systematic review and meta-analysis. Cureus. (2024) 16:e59855. doi: 10.7759/cureus.59855, 38854278 PMC11162285

[9] Ghatge SB Shah RP Surya N Sankhala S Unadkat CJ Khan GM . Ozone disc nucleolysis in cervical intervertebral disc herniation: a nonrandomized prospective analysis in 246 patients. J Craniovertebr Junction Spine. (2022) 13:114–20. doi: 10.4103/jcvjs.jcvjs_46_22, 35837424 PMC9274671

[10] Korecki CL Costi JJ Iatridis JC. Needle puncture injury affects intervertebral disc mechanics and biology in an organ culture model. Spine (Phila Pa 1976). (2008) 33:235–241. doi: 10.1097/BRS.0b013e3181624504 18303454 PMC2587060

[11] Jain C Huang JJ Lee Y Chaudhary S Hecht AC Lai A . Animal models of disc degeneration using puncture injury: a 20 year perspective. JOR Spine. (2025) 8:e70093. doi: 10.1002/jsp2.70093, 40727550 PMC12301940

[12] Michalek AJ Buckley MR Bonassar LJ Cohen I Iatridis JC. The effects of needle puncture injury on microscale shear strain in the intervertebral disc annulus fibrosus. Spine J. (2010) 10:1098–105. doi: 10.1016/j.spinee.2010.09.015, 20971041 PMC2991597

[13] Ma Y Yu X Li W Guan J Qiu Z Xu L . Animal models of internal endplate injury-induced intervertebral disc degeneration: a systematic review. J Investig Surg. (2024) 37:2400478. doi: 10.1080/08941939.2024.2400478, 39255967

[14] Lipetz JS. Pathophysiology of inflammatory, degenerative, and compressive radiculopathies. Phys Med Rehabil Clin N Am. (2002) 13:439–49. doi: 10.1016/s1047-9651(02)00005-0, 12380544

[15] Celenlioglu AE Solmaz I Eksert S Simsek F Ilkbahar S Sir E. Factors associated with treatment success after interlaminar epidural steroid injection for cervical radicular pain. Turk Neurosurg. (2023) 33:326–33. doi: 10.5137/1019-5149.JTN.42539-22.2, 36799281

[16] Delgado DA Lambert BS Boutris N McCulloch PC Robbins AB Moreno MR . Validation of digital visual analog scale pain scoring with a traditional paper-based visual analog scale in adults. J Am Acad Orthop Surg Glob Res Rev. (2018) 2:e088. doi: 10.5435/JAAOSGlobal-D-17-00088, 30211382 PMC6132313

[17] Widbom-Kolhanen S Pernaa KI Saltychev M. Reliability and validity of the Neck Disability Index among patients undergoing cervical surgery. Int J Rehabil Res. (2022) 45:273–8. doi: 10.1097/MRR.0000000000000540, 35776945

[18] Takahashi N Yabuki S Aoki Y Kikuchi S. Pathomechanisms of nerve root injury caused by disc herniation: an experimental study of mechanical compression and chemical irritation. Spine. (2003) 28:435–41. doi: 10.1097/01.BRS.0000048645.33118.02, 12616153

[19] Ye F Lyu FJ Wang H Zheng Z. The involvement of immune system in intervertebral disc herniation and degeneration. JOR Spine. (2022) 5:e1196. doi: 10.1002/jsp2.1196, 35386754 PMC8966871

[20] Sun Z Liu B Luo ZJ. The immune privilege of the intervertebral disc: implications for intervertebral disc degeneration treatment. Int J Med Sci. (2020) 17:685–92. doi: 10.7150/ijms.42238, 32210719 PMC7085207

[21] Bocci V Borrelli E Zanardi I Travagli V. The usefulness of ozone treatment in spinal pain. Drug Des Devel Ther. (2015) 9:2677–2685. doi: 10.2147/DDDT.S74518 26028964 PMC4440430

[22] Hidalgo-Tallón FJ Torres-Morera LM Baeza-Noci J Carrillo-Izquierdo MD Pinto-Bonilla R. Updated review on ozone therapy in pain medicine. Front Physiol. (2022) 13:840623. doi: 10.3389/fphys.2022.840623, 35283802 PMC8904924

[23] Erario MLÁ Croce E Moviglia Brandolino MT Moviglia G Grangeat AM. Ozone as modulator of resorption and inflammatory response in extruded nucleus pulposus herniation. Revising concepts. Int J Mol Sci. (2021) 22:9946. doi: 10.3390/ijms22189946 34576108 PMC8469341

[24] Alorfi NM. Pharmacological methods of pain management: narrative review of medication used. Int J Gen Med. (2023) 16:3247–56. doi: 10.2147/IJGM.S419239, 37546242 PMC10402723

[25] Piacherski V Marachkou A. Pharmacological and clinical implications of local anaesthetic mixtures: a narrative review. Anaesthesia. (2022) 77:939. doi: 10.1111/anae.15726, 35319095

[26] Lutz G. Infections following interventional spine procedures: a systematic review. Pain Physician. (2021) 24:101–16. doi: 10.36076/ppj.2021.24.101-116 33740341

[27] Rawlings CE Wilkins RH Gallis HA Goldner LJ Francis R. Postoperative intervertebral disc space infection. Neurosurgery. (1983) 13:371–6. doi: 10.1227/00006123-198310000-00004, 6633829

[28] Xiao X Wei Z Ren H Sun H Luo F. Comparison of effectiveness and safety between intraoperative 3D-CT-guided and C-arm-guided percutaneous balloon compression for idiopathic trigeminal neuralgia: a multi-center retrospective study. Pain Res Manag. (2021) 2021:9306532. doi: 10.1155/2021/9306532, 34194588 PMC8203368

